# Low-cost air pollution monitoring system—an opportunity for reducing the health risk associated with physical activity in polluted air

**DOI:** 10.7717/peerj.10041

**Published:** 2020-10-01

**Authors:** Zenon Nieckarz, Jerzy A. Zoladz

**Affiliations:** 1 Experimental Computer Physics Department, Marian Smoluchowski Institute of Physics, Jagiellonian University, Krakow, Poland; 2 Department of Muscle Physiology, Institute of Basic Sciences, Faculty of Rehabilitation, University School of Physical Education, Krakow, Poland

**Keywords:** Particulate matter, Air pollution, Physical activity, Health, Sensors

## Abstract

The issue of air pollution by particulate matter (PM) concerns many places in the world. At the same time, many residents undertake physical activity (recreation, rehabilitation, sport) in the open air. Generally, the amount of dust concentration depends on both the place (center or periphery of the city) and the time of day. In the present study we describe the outcome of monitoring of the state of air pollution by particle matter (PM_10_) in the Kraków agglomeration area in order to show that it can provide information concerning air quality in the area where people practice varied kinds of sports in the open air. The measurements of PM_10_ have been made by a few stations with identical construction working as one network. The details of the air pollution monitoring system and its data quality verification have been described. The network stations made multipoint observations across the Kraków Metropolitan Area during the year 2017 in eight locations. The locations selected represent a diverse spectrum of terrain conditions in which the Kraków agglomeration community undertakes physical activity. For most months of 2017, the minimum monthly average 4-hour PM_10_ concentrations were recorded between 10–14 h, regardless of location, whereas the maximum was between 18–22. We also noticed a huge differences in the average monthly value of PM_10_ in some locations within the Kraków agglomeration—ranging between 4.9–339.0 µg m^−3^. This indicates that some regions of the city are more suitable for performance of physical activity in the open air than others. In conclusion, we postulate that a low-cost air pollution monitoring system is capable of providing valuable information concerning air quality in a given region, which seems to be of importance also to people who practice varied sports activities in the open air.

## Introduction

The presence of high concentrations of particulate matter (PM) in the air is an issue that concerns everybody globally (WHO, 2006). It is well documented that exposure to high PM on a short-term scale (Levy, Hammitt & Spengler, 2000; Nawrot et al., 2011; Provost et al., 2016) and in the long term (Beelen et al., 2014) increases the risk of many serious diseases, including cardiovascular and respiratory diseases (Du et al., 2016; Jo et al., 2017) especially in children (Saenen et al., 2016) and the elderly (Halonen et al., 2009; Simoni et al., 2015). A topic which has now been receiving special attention is the impact of air pollution on the health of physically active people (An et al., 2017; Giles & Koehle, 2013) practicing sports in the open air, including athletes (Kuskowska, Rogula-Kozłowska & Widziewicz, 2019; Reche et al., 2020).

Unfortunately, high PM concentrations are increasingly observed in the vast urban areas of many cities around the world, e.g., Delhi, Causeway Bay in Hong Kong (Cropper et al., 1997; Gupta et al., 2006; Jedrychowski et al., 2006) and others. Factors strongly increasing air pollution in cities include thermal inversions (Czarnecka & Nidzgorska-Lencewicz, 2017) and poor ventilation.

In Poland, a prime example of a city struggling with high air pollution resulting, among others, from the low ventilation ratio is the city of Kraków (Bokwa, 2008; Jedrychowski, 2007; Osrodka et al., 2010; Piotrowicz, 2010). According to a recent study by Wilczyńska-Michalik & Michalik (2017), the principal sources of PM in Kraków are: vehicle emission; so-called low-emission—related to household heating; emission from industrial plants and numerous small factories; resuspension from streets, construction and demolition sites; resuspension from soil; and dispersed plant debris. The most dominant components of air pollution in Krakow are PM, benzo(a)pyrene in PM, and nitrogen oxides, but other components—such as soot, which dominates in PM_2.5_, and polycyclic aromatic hydrocarbons—are also present in substantial quantities. The highest PM_2.5_ concentrations related to concentrations of polycyclic aromatic hydrocarbons are normally observed during summer and winter seasons as a result of industrial and traffic pollution, and the seasonal low-emission (i.e., emission generated by household heating, industrial plants and numerous small factories) (Samek, Stegowski & Furman, 2016).

Kraków has a large population, currently over 771 thousand (Anon, 2019) and many of the inhabitants practice physical activity in varied forms (including running, cycling, and/or walking) in the open air, during different times of the day or night.

Physical activity, due to its well documented positive impact on varied systems of the human body (for an overview see, e.g., Kemmler & Von Stengel, 2019; Pedersen & Saltin, 2015; Wang, Gao & Zucker, 2019), is recommended by various respected international organizations, such as the World Health Organization (WHO, 2010), the European Commission (EC, 2013), the Center for Disease Control and Prevention (CDCP, 2020) and the American College of Sports Medicine (Pate et al., 1995), to be practiced regularly by people, regardless of age. However, it should be realized that any form of physical activity increases the amount of air ventilated by the lungs (minute ventilation—*V*_*E*_), which is a few times higher during exercise even of moderate intensity than when at rest (see e.g., Åstrand & Rodahl, 1986; Bowen, Benson & Rossiter, 2019; Zoladz, Grassi & Szkutnik, 2019). An increase of the *V*_*E*_ enhances the inflow of varied PM into the lungs and increases their deposition in the respiratory tract. Another factor that influences the PM deposition rate in the respiratory tract of exercising people is the quality of the air, namely the amount of varied PM in the air. Unfortunately, the issue of the impact of air pollution on people’s health, especially those who practice physical exercise, is largely ignored.

In recent years, the possibility of constructing low-cost PM sensors attracted the attention of many researchers around the world (Johnston et al., 2019; Chojer et al., 2020). Most of the research has been focused on the following issues: (i) evaluation and calibration of various PM sensor systems (Wang et al., 2015; Bulot et al., 2019; Markowicz & Chiliński, 2020); (ii) detection of local sources of air pollution (Kumar et al., 2015; Morawska et al., 2018; Rogulski, 2018); (iii) influence of meteorological and topographic parameters on air pollution (Gao, Cao & Seto, 2015; Rogulski, 2017); and (iv) studies aimed at evaluating mortality risk associated with exposure to air pollution (Castell et al., 2017; Chen et al., 2017). To the best of our knowledge, the present paper is the first attempt to draw the attention of a potential readers to potential applications of low cost PM systems that could be considered as a part of the sports infrastructure and allow the benefits of physical exercise practiced in the open air to be enhanced. This can be done by using the system to continuously provide information about air pollution in the zone where physical activities are practiced, which would allow people to adjust the length and intensity of their activities, according to the air quality on a given day.

This is why, in the present study, we analyzed the state of air pollution by particle matter (PM_10_) in the Kraków agglomeration area in order to assess the health hazard of physical activity performed in this city. The study was conducted using the measurements made by a few stations with identical constructions and the same verified accuracy of measurements. In Section 2 we have described the air pollution monitoring system and its data quality verification. In Section 3, we have presented the stations’ distribution in space during multipoint observations, as well as the results of measurements and analyses. Section 4 contains our conclusions.

## Materials and Methods

In the present paper, a low-cost air pollution monitoring system is described as a useful tool for limiting the health hazard of physical activity performed in polluted air. Although our dust sensor was capable of measuring PM_10_, PM_2.5_, and PM_1.0_, in the present study we presented only the data related to PM_10_ and PM_2.5_. In this paper, we decided not to present the PM_1_-related data, since we were still unable to verify their quality.

The construction, mode of operation, and accuracy are presented in the next subsections.

### General information

As part of the scientific project, known as the Storm&DustNet implemented at the Jagiellonian University in Kraków (Poland), the Jagiellonian University Network of Stations (JUNS) was created, dedicated to monitoring the concentration of PM in the atmosphere, as well as selected meteorological parameters (including air temperature, humidity, and atmospheric pressure). The first stations began working under JUNS at the beginning of 2016.

The central element of JUNS is a data server with software written in the script programming language Python. This server receives data from measuring stations and allows the administrator full access to data, as well as remote diagnosis of the technical condition of individual university measuring stations (UMS), which usually work far in the field. The data server processes the data flowing from individual stations on an ongoing basis, prepares the necessary information to be displayed on the website, and places it on the web-server. As a result, every Internet user can observe the results of the online measurements in the public domain via a website (http://inhalation.uj.edu.pl).

**Figure 1:**
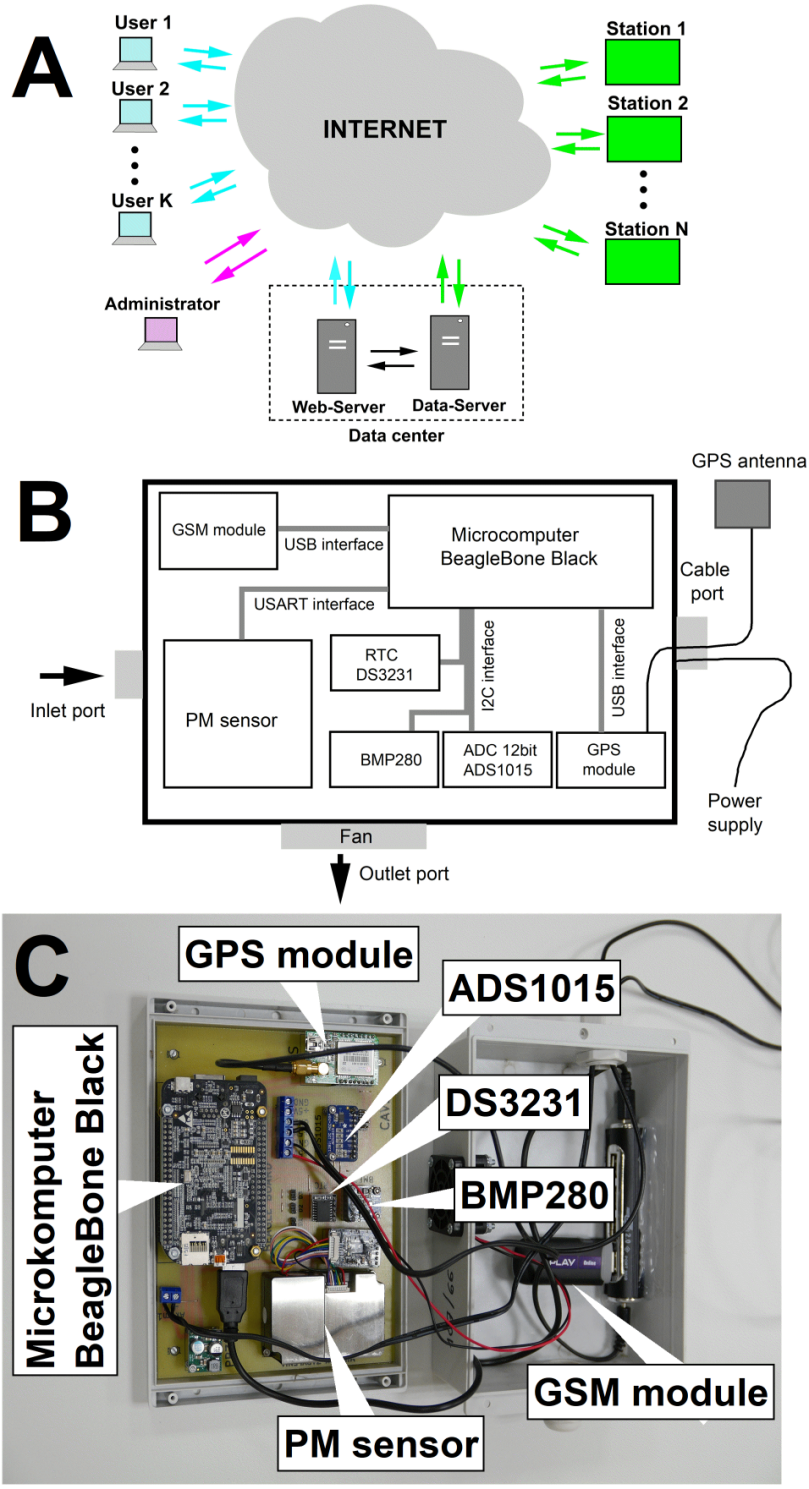
Diagram of the measuring network (A), block diagram of the station (B) and view (photo) of the arrangement of elements inside the station (C).

The general schematic diagram of JUNS is presented in Fig. 1A. The network is built in the star topology, and each measuring station connects to the data server via the Internet using one of two techniques: (a) wireless transmission using module GSM/UMTS/LTE; or (b) standard cable connection TCP/IP. The JUNS network architecture ensures easy scalability of the solution in both the hardware and software layers.

### Characteristic of servers

Both servers included in JUNS are embedded in the technical Internet infrastructure of the Faculty of Physics, Astronomy, and Applied Computer Science of the Jagiellonian University, and work on the basis of the Linux system (version SUSE 4.4.162). Communication of the measuring stations with the data server is carried out by cryptographic network protocol (SSH), and the results of the measurements are stored in the MySQL relational database. All access to the measurement results is therefore made through the query language of the SQL Server Database.

### Characteristics of measuring stations

The university measuring stations (UMS) have a uniform structure and software written in Python. The components of each of them are: (a) minicomputer BeagleBone Black (Arrow Electronics, USA), which is a low cost and popular development board for developers and the avid hobbyist; (b) wireless communication module GSM E3131h-2 (Huawei, China); (c) digital laser dust sensor SEN0177 (DFRobot, China) which measures the particle concentration of suspended particulate matter in the air; (d) integrated humidity, temperature, and pressure sensor BME280 (Bosch, Germany); (e) precision real-time clock module DS3231 (Maxim Integrated, USA); (f) module GPS MIKROE-1032 (MikroElektronika, Serbia); (f) analog-digital converter ADS1015 (Texas Instruments, USA), which is used to monitor the input supply voltage of the station and 3.3V in the internal power bus; (g) step-down voltage converter D24V50F5 (Pololu, USA), which enables DC power supply in from 6.0 to 38.0 V by a standard power supply or accumulator. The average power consumption of the station is equal to 5.4 W (450mA@12V). The quality of measurements made with the DFRobot sensor has been confirmed in former studies (Rogulski, 2017; Markowicz & Chiliński, 2020).The block diagram of the station is presented in Fig. 1B, and the view of the arrangement of the components in the housing of the measuring station is shown in Fig. 1C. According to the technical specifications of the manufacturers, all components of the measuring stations can be operated in the humidity range of 5–95% and the temperature from −20 °C to 50 °C. The external dimensions of the station are 20 × 15 × 7.3 cm (LxWxH), and the weight is 0.6 kg. The station can work as a stationary or mobile device. The total cost of building the station was approximately 400 USD, estimated at the beginning of 2020.

### Data quality

The station measured the concentration of particle matter and air temperature, humidity, and pressure several dozen times per minute. The parameters were averaged over one minute intervals. Then, the average values were sent to the data server and saved to a database. Measurements of the air temperature, relative humidity (RH) and pressure were taken using a BME280 detector installed inside the UMS (temperature range: −40 to +85 °C with accuracy: ±1 °C; humidity rage: 10 to 80% RH with accuracy: ±3% RH; pressure range: 300 to 1100 hPa with accuracy: ±1 hPa).

### Laboratory verification of PM_10_ data quality

Although UMS measures three indices (PM_10_, PM_2.5_ and PM_1_), we verified the most popular PM_10_ mass index for which was determined in Poland a daily permissible level of 50 µg m^−3^ (Chief Inspectorate For Environmental Protection, 2015) . The UMS’s final accuracy of the concentration measurements of suspended particulate matter PM_10_ was determined on the basis of test measurements carried out for this purpose. Results recorded by UMS have been compared with data obtained from the reference analyzer EDM107 produced by GRIMM (GRIMM Aerosol Technik, Germany). Both apparatus were operated in the same place during the test. The EDM107 was our calibration standard of known accuracy, with the measurement error amounting to ±2 µg m^−3^. This analyzer has a certificate of calibration and equivalency to a gravimetric method (Grimm & Eatough, 2009) .

Quality verification of data was carried out simultaneously for 15 UMS stations, which were placed side by side with the analyzer EDM107 in the laboratory next to the window (about 1 m away). The distance between the air inlet and the analyzer, and between the air inlet and each UMS station, did not exceed 50 cm. One additional UMS station (named St4) worked outside the building near the lab window.

**Figure 2:**
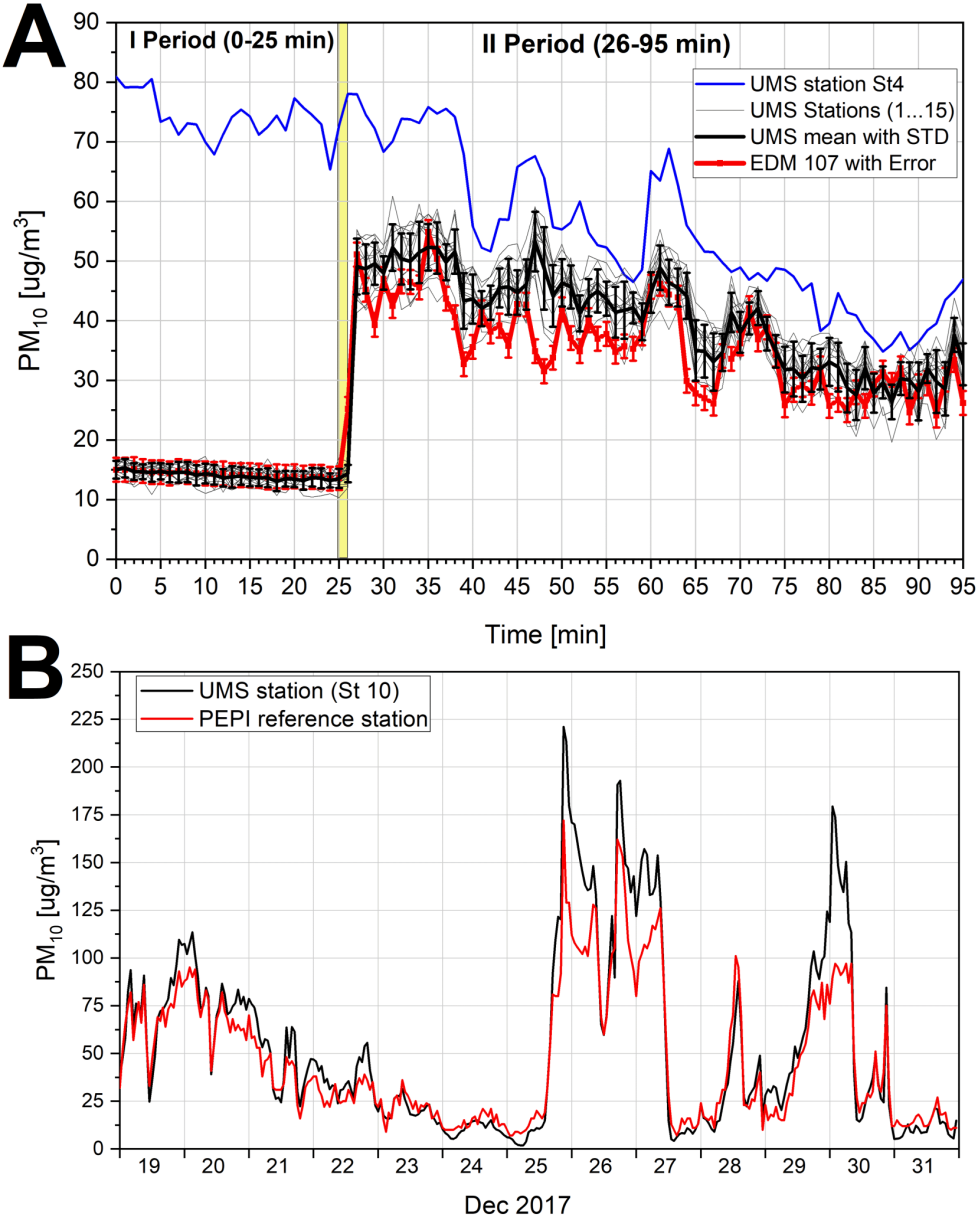
(A) The time distribution of PM_10_ mass concentration recorded during 95 minutes of tests. (B) The distribution of the mean hourly PM_10_ measured by stations St10 and the validated station. (A) The time distribution of PM10 mass concentration recorded during 95 minutes (07:10–08:45) of tests by the EDM107 analyzer (red line with error bars for accuracy), fifteen UMS stations (thin black lines), their mean (thick black line with error bars for standard deviation) and the additional UMS station (St4) installed outside the laboratory in open air (blue line). The synchronic sampling of data was one minute for all instruments. Period I—represents the records performed in the laboratory room with closed windows and Period II—represents the measurements conducted in the same laboratory, but with an open window. (B) The distribution of the mean hourly PM_10_ measured by stations St10 (black color) and the validated monitoring station belonging to the Provincial Environmental Protection Inspectorate (Kraków, Poland), which operated in Skawina (red color) from 19 to 31 December 2017.

The results of the comparative test measurements is presented in Fig. 2A, where the thin black lines represent data recorded by 15 UMSs, the bold black line is the average from measurements made by 15 UMSs and standard deviation; the bold red line represents data recorded by the analyzer EDM107 and its error; and the blue line represents data recorded by the additional station St4.

The comparative measurements lasted 95 min (07:10-08:45) and consisted of two periods. The first period lasted 25 min (07:10-07:35) and the window was closed in the laboratory which had clean air. All UMSs registered low concentrations of PM_10_, andthe mean values were 14.0 µg m^−3^ (SD = ±1.5 µg m^−3^). The EDM107 analyzer recorded a mean of 14.2 µg m^−3^ (SD = ±0.5 µg m^−3^).

During the second period, the window was completely open (07:36-08:45). After opening the window, the polluted air from outside flowed into the laboratory. The window opening time and the rapid influx of polluted air are marked with the yellow rectangle in Fig. 2A. During the comparative measurement, the PM_10_ values recorded individually by 15 UMSs did not differ more than ±10 µg m^−3^ from the results recorded by the EDM107 analyzer. Time distributions of the PM indicator registered by each UMS station correlated with the results recorded by EDM107 (Spearman coefficient in range 0.86–0.94).

In summary, the comparative measurements described above included natural ambient air and various particulate matter concentrations. As a result, the comparison showed that measurements of PM_10_ made by UMS stations are encumbered with an average error that does not exceed ±10 µg m^−3^.

### Practical verification of PM_10_ data quality

The other method of verification was the comparison of measurements made by the validated monitoring station belonging to the Provincial Environmental Protection Inspectorate (PEPI) in Kraków, and the results made by one of the UMS stations (St10), which operated together in this same place and time. Comparative measurements were taken in the time period 19-31.12.2017 in Skawina, in close proximity to the city of Kraków. The distribution of hourly averages of particulate matter PM_10_ measured by both stations is presented in Fig. 2B, and the correlation coefficient was very high (*R* = 0.96). The average value of PM_10_ calculated based on the measurements taken by the St10 station was 15% higher than the average calculated on the basis of the measurements taken by the validated station PEPI. As can be seen in Fig. 2B, the results of measurements from the St10 station are higher than those of the PEPI station mainly for PM_10_ greater than 100 µg m^−3^. For PM_10_ below 100 µg m^−3^, the average error for the St10 station does not exceed 9 µg m^−3^.

### Summary of the PM_10_ data quality verification

The comparative analysis of the PM_10_ data quality recorded by UMS stations using two approaches was completed. In the data range below 100 µg m^−3^, both the laboratory and the practical analysis gave the comparative inaccuracy equal ±10 µg m^−3^ and ±9 µg m^−3^. Considering the wider range of data (above 100 µg m^−3^), the UMS stations made measurements with an accuracy of 15%.

### The spatial distribution of the JUNS

**Figure 3:**
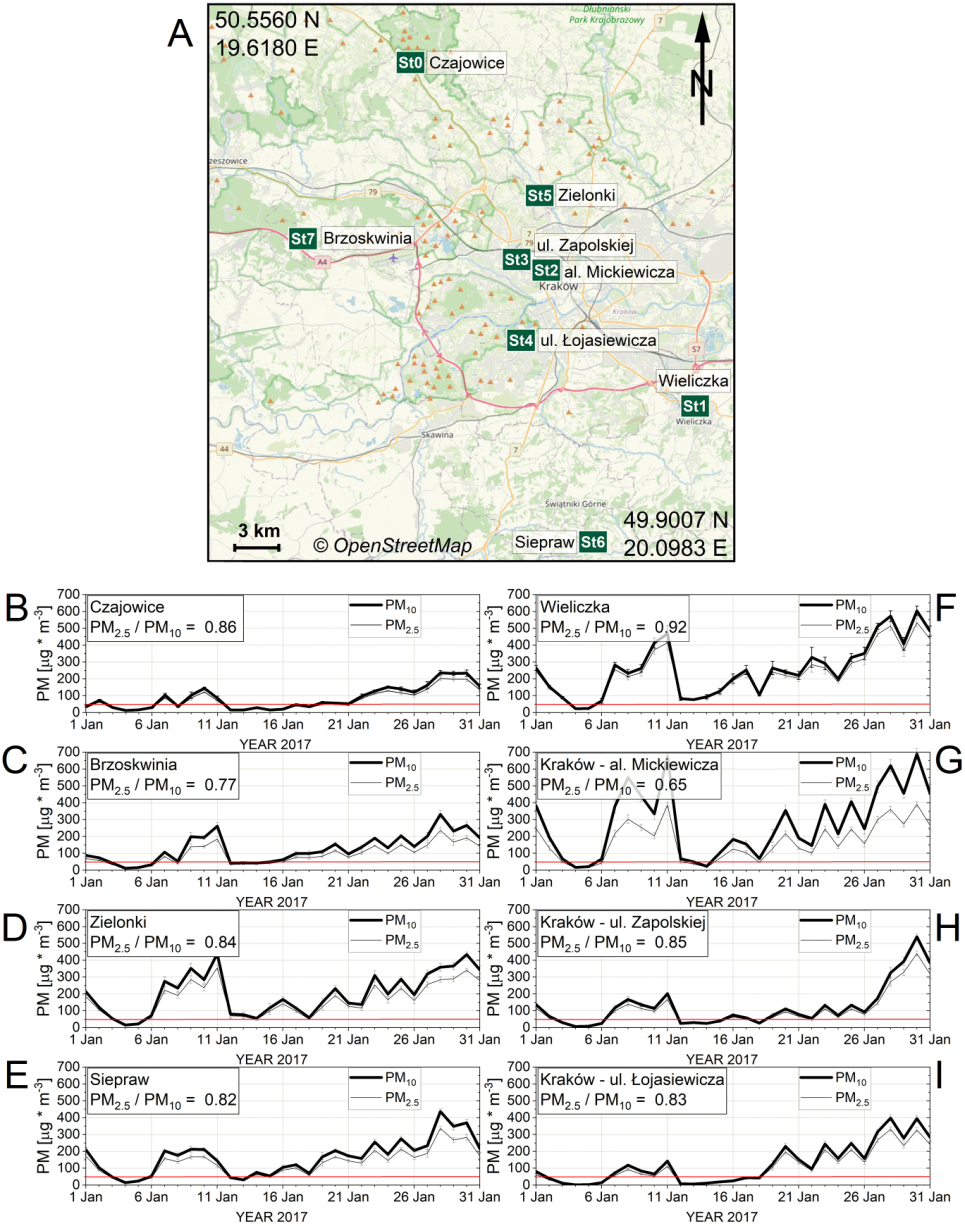
(A) Location map of measuring stations, developed using OpenStreetMap (CC BY-SA 4.0). (B-I) The distribution of average daily PM_10_ and PM_2.5_ values recorded in January 2017 by stations. (A) The location of the station is marked by the green rectangles with the inscription “St” together with the station number from 0 to 9: St0, Czajowice; St1, Wieliczka; St2, Kraków-al. Mickiewicza; St3, Kraków-ul. Zapolskiej; St4, Kraków-ul. Łojasiewicza; St5, Zielonki; St6, Siepraw; St7, Brzoskwinia. (B–E) The distribution of average daily PM_10_ and PM_2.5_ values recorded in January 2017 by stations installed in the villages: Czajowice, Brzoskwinia, Zielonki, Siepraw. (F–I) The distribution of average daily PM_10_ and PM_2.5_ values recorded in January 2017 by stations installed in the town Wieliczka and three localizations in Kraków: al. Mickiewicza, ul. Zapolskiej, ul. Łojasiewicza. The red horizontal line represents the daily permissible level 50 µg m^−3^.

The JUNS made their first multipoint observations over the Kraków Metropolitan Area during 2017 in 8 localizations, as presented in Fig. 3A. Locations were selected that represent a diverse spectrum of terrain conditions in which the Kraków agglomeration community undertakes physical activity. Therefore, the types of buildings, traffic intensity, and the presence of vegetation were taken into account. The UMS stations were installed mainly on buildings, at a height around 2.5 m above the ground. Four stations were installed in villages close to Kraków, where the building density is low: Czajowice (St0), Brzoskwinia (St7), Zielonki (St8), and Siepraw (St9). One station monitored air quality in the town Wieliczka (St1) next to Kraków, and three stations monitored air quality in the city of Kraków: al. Mickiewicza (St2), one of the main streets of the city; ul. Zapolskiej (St3), a housing estate of compact houses; ul. Łojasiewicza (St4), the Jagiellonian University Campus, which is close to green areas.

It should be mentioned that some of our measurement stations belonging to the JUNS were placed in the regions of the Krakow Metropolitan Area, where people used to practice varied forms of outdoor physical activities. For example, station (St2) in al. Mickiewicza is located close to the Planty Krakowskie park area and to the eastern part of the grassland zone called Błonia Krakowskie. Similarly, the station (St1) in Wieliczka is located very close to the athletics stadium, where sportsmen practice varied sports and participate in competitions. As can be seen in Fig. 3A, popular locations chosen by the people to practice varied outdoor physical activities - such as Planty Krakowskie and the eastern part of the zone Błonia Krakowskie, located close to station (St2) in al. Mickiewicza (Fig. 3A), as well as the athletics stadium in Wieliczka (St1), are unfortunately characterized by poorer air quality than the other locations studied within our project (see e.g., (St0) Czajowice - a village situated just on the border of the town).

## Results

### Choosing a place for physical activity during the winter (or cold season) through the lens of air quality

It should be noted that physical activity is undertaken in different seasons in the year, both in warmer and colder months. Unfortunately, in winter, as a result of low temperatures and increased combustion, as well as unfavorable meteorological situations (no wind) (Scibor, Bokwa & Balcerzak, 2020), there are episodes of high concentrations of particulate matter which pose a real threat to human health.

In 2017, particularly long periods of high air pollution were recorded in January. In all eight monitored locations, PM_10_ exceeded the permissible level (50 µg m^−3^) on most days of January. The largest number of days exceeding the permissible level occurred in Wieliczka (29), and the smallest in Czajowice (17). The average monthly value of PM_10_ for January 2017 was the highest in Kraków-al. Mickiewicza (289 µg m^−3^), and the lowest in Czajowice (86 µg m^−3^). The highest daily PM_10_ concentration occurred on 30.01.2017, when the stations recorded maximal PM_10_ values in Kraków-al. Mickiewicza (696 µg m^−3^), as well as high levels in Wieliczka (605 µg m^−3^). The monthly average values of PM_10_ recorded by stations in rural areas were in the range of 86–203 µg m^−3^, and the distribution of daily values in January 2017 is presented in Fig. 3 (panels B, C, D, E). The monthly average values of PM_10_ recorded by stations in urban areas were in the range of 126-289 µg m^−3^, and the distribution of daily values in January 2017 is presented in Fig. 3 (panels F, G, H, I). The PM_2.5_/PM_10_ ratio observed in our study was 0.82, which is very close to the values previously reported by others (Wilczyńska-Michalik & Michalik, 2017).

### Example of choosing the optimal place and time for open-air physical activity in the future

**Figure 4:**
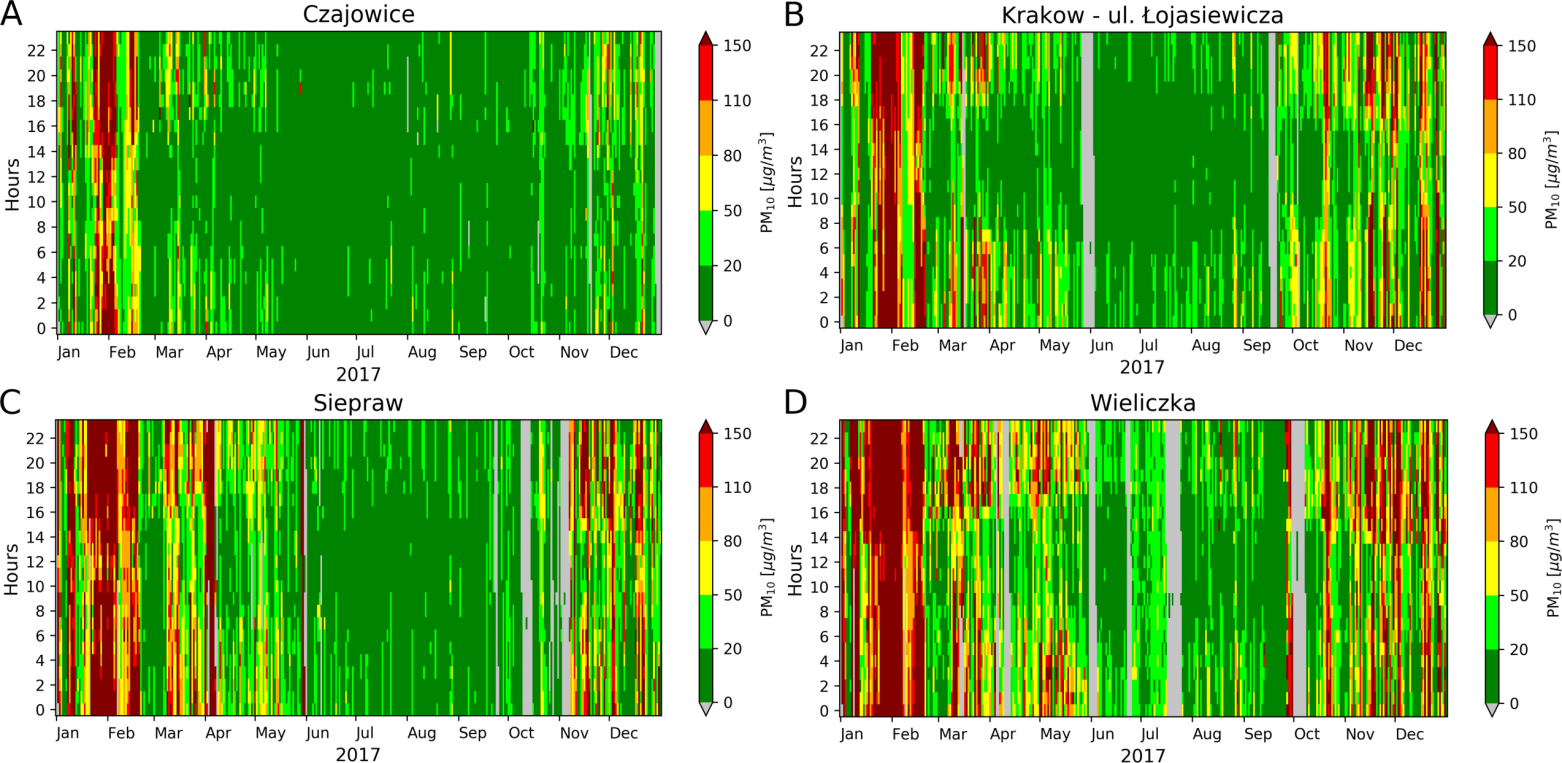
The color-coded distribution of the hourly PM_10_ vs number of days of the year 2007 using Air Quality Levels are presented as a color bar. In contrast, a gray background indicates a time when no observations were carried out. Results in A, B, C, D for localizations vis Czajowice, Kraków-ul. Łojasiewicza, Siepraw and Wieliczka.

The concentration of PM_10_ depends on several factors, such as location, season, and time of day. All of them have their basis in landform, weather conditions, economic activities, and daily human life. Current decisions about the choice of place and time of physical activity in the open air can be made on the basis of available air quality measurements. However, in long-term planning, e.g., for future major sporting events, it is good to make a decision after reviewing the archival results of air quality measurements in the considered locations. Figure 4 presents examples of results recorded in 2017 by four select stations, for which an example analysis was made. The distribution of PM_10_ concentration as a function of time of day and season is color-coded. Vertical areas of red shades are clearly visible, and correspond to periods of several days in which the stations recorded very high concentrations of PM_10_ occurring 24-hours per day and day by day. During this time, the average hourly PM_10_ values were over 80 µg m^−3^ (colors: orange, red and burgundy) exceeding around twice (or more) of the daily limit value (50 µg m^−3^).

In 2017, the minimum and maximum value of the average PM_10_ concentration in the examined hour occurred between 10:00–14:00 and 18:00-22:00, regardless of location (Table 1), the minimum value was in village Czajowice (20.6 µg m^−3^), and the maximum in Wieliczka (120.1 µg m^−3^). At all locations, the largest percentage of time with PM_10_ with a Good Air Quality Level (AQL) occurred between 10:00–14:00 (maximum 70% in Czajowice), and the lowest between 18:00–22:00 (minimum 11% in Wieliczka). The biggest percent of time with Extremely Poor AQL were between 18:00–22:00, independent of location (maximum 25% in Wieliczka).

### Minimizing the health risks from exposure to air pollution

Minimizing the health risks from exposure to air pollution on an annual scale can be determined using the results in Table 2. For most months of 2017, minimum monthly average 4-hour PM_10_ concentrations were recorded between 10:00–14:00 regardless of location. In general, the minimum average monthly value of PM_10_ was recorded between 14:00–18:00 in Czajowice (4.9 µg m^−3^), and the maximum between 18:00–22:00 in Wieliczka (339.0 µg m^−3^).

It should be emphasized that the results presented in Fig. 4 come from locations of different natures and degrees of urbanization. Czajowice and Siepraw are definitely rural areas, Wieliczka is a small town, and the area of the III Campus of the Jagiellonian University represents the edge of the recreation area in the southwest of Kraków. The biggest area of green color in Fig. 4A indicates good and very good Air Quality Level, which was in Czajowice.

The results showed that, among the four locations selected, the village Czajowice is most often the best choice for physical activity in the open air. In contrast, as seen in Fig. 4D, the Wieliczka area is characterized by frequent periods with elevated PM_10_ concentrations, indicating that this is not a suitable place for activity in the open air.

**Table 1:** Mean value of PM_10_ and percent of time for the selected periods (06:00–10:00, 10:00–14:00, 14:00–18:00, 18:00–22:00) in six air quality levels in 2017.

Station designation and localization	Hours	Mean PM_10_ [µg/m^3^]	Percent of Time in Air Quality Level (AQL)					
			Good <20 [µg/m^3^]	Fair 20–50 [µg/m^3^]	Moderate 50–80 [µg/m^3^]	Poor 80–110 [µg/m^3^]	Very Poor 110–150 [µg/m^3^]	Ext. Poor >150 [µg/m^3^]
St0—Czajowice	06–10	21.6	67	23	5	2	2	1
	10–14	20.6	70	21	4	2	2	1
	14–18	29.4	62	23	6	3	2	4
	18–22	36.7	50	32	7	4	3	4
St1—Wieliczka	06–10	75.6	25	35	12	8	7	13
	10–14	59.2	36	29	13	7	6	9
	14–18	94.4	25	29	11	8	8	19
	18–22	120.1	11	29	15	9	11	25
St4—Kraków ul. Łojasiewicza	06–10	49.3	47	26	10	6	3	8
	10–14	31.3	61	22	7	4	3	3
	14–18	37.6	55	23	9	4	5	4
	18–22	60.4	36	28	13	7	6	10
St6—Siepraw	06–10	54.4	43	28	10	5	5	9
	10–14	45.5	51	22	10	5	5	7
	14–18	60.1	42	23	12	5	6	12
	18–22	77.8	25	33	11	9	5	17

**Table 2:** Monthly mean value of the PM_10_ for the selected four-hour periods (06:00–10:00, 10:00–14:00, 14:00–18:00, 18:00–22:00) in 2017.

Localization	4-hour period	2017											
		Jan	Feb	Mar	Apr	May	Jun	Jul	Aug	Sep	Oct	Nov	Dec
St0—Czajowice	06–10	62.3	49.8	21.9	14.7	12.6	7.2	9.1	11.1	9.6	12.9	21.7	29.1
	10–14	71.2	49.4	14.9	13.8	10.7	5.2	7.2	9.9	8.4	11.3	18.9	28.5
	14–18	107.8	72.1	24.2	15.6	11.5	4.9	7.0	11.9	11.7	19.2	37.3	33.3
	18–22	114.0	79.8	44.9	28.7	18.9	8.1	12.3	16.9	11.8	21.9	40.9	44.4
St1—Wieliczka	06–10	218.0	170.9	84.4	50.5	46.5	23.1	27.6	23.7	25.0	46.4	84.9	83.3
	10–14	184.2	123.1	52.5	33.8	35.9	18.8	28.1	17.7	14.6	38.1	70.2	72.8
	14–18	294.8	188.2	86.7	48.2	40.3	21.4	27.7	23.4	19.7	74.0	143.9	121.0
	18–22	339.0	224.7	161.1	96.1	90.3	33.6	33.2	40.0	28.4	76.7	147.5	116.5
St4—Kraków ul. Łojasiewicza	06–10	126.4	110.9	55.4	25.3	27.3	10.6	11.4	18.4	23.4	36.0	71.7	68.5
	10–14	87.8	74.8	25.9	12.8	17.8	5.4	6.5	11.6	16.6	23.2	39.7	49.5
	14–18	99.4	87.0	26.1	15.6	16.9	6.2	6.3	11.1	20.5	35.5	59.6	61.7
	18–22	143.8	115.7	71.2	36.7	27.3	12.7	15.0	21.6	29.0	59.5	98.7	86.1
St6—Siepraw	06–10	143.6	124.0	78.1	51.1	41.3	18.9	13.2	15.0	15.1	19.6	64.9	65.3
	10–14	127.4	97.7	50.7	52.9	35.0	12.2	10.0	12.5	14.7	17.4	52.3	60.1
	14–18	178.9	125.9	55.1	57.8	43.1	13.3	11.1	13.0	16.1	27.9	91.3	93.2
	18–22	203.9	156.5	105.0	80.1	57.1	23.1	19.6	20.5	20.0	30.5	115.3	96.4

Despite the important general differences in locations of the UMS stations, short periods of high PM_10_ concentrations are clearly visible at all locations (Fig. 4), although with varying intensity. This indicates the large spatial coverage of this phenomenon covering at least the area of the Kraków agglomeration. In this case, the recommendation should be that the all unnecessary outdoor activities should be abandoned.

## Discussion

### Possible applications of the system

In recent years, one has witnessed growing popularity of low-cost air pollution monitoring systems and their applications in several fields. Simple low-cost systems can track short-lived pollution events (Bulot et al., 2019), monitor pollution in smart cities (Chen et al., 2017; Johnston et al., 2019), provide aggregated information about observed air quality outdoors (Castell et al., 2017; Rogulski, 2018), in flats and houses and in the zoological garden area (Pawlak & Nieckarz, 2020). Generally, it is recommended that a credible validation of low-cost sensors in a future study is performed (Chojer et al., 2020).

As shown above, simple systems, such as the unit used in the present study, can be used to monitor and register the quality of air, which could firstly be applied to the industrial areas of densely populated parts of a country. It could be also suitable in villages, especially those offering an attractive touristic service. Finally, this system could be valuable for monitoring air conditions in parks and sports areas attracting a large amount of people who practice various kinds of open air activities.

Knowledge concerning the quality of air in a given place might have a pivotal role in planning the duration of time to spend in the open air, as well as for planning the amount and intensity of training for a given person on a certain day. This seems to be especially important since any form of sustained exercise (including physical labor) increases the body’s need for oxygen, which enhances the amount of the air ventilated by the human lungs (for an overview see, e.g., Bowen, Benson & Rossiter, 2019; Wasserman et al., 2011; Zoladz, Grassi & Szkutnik, 2019). For example the minute ventilation (*V*_*E*_) which amounts to about 6–8 liters of air for humans at rest, increases to 30–50 liters per minute during exercise of moderate intensity, and exceeds 100 L per minute during strenuous exercise (Wasserman et al., 2011). In some athletes, maximal *V*_*E*_ during strenuous exercise can exceed 200 L per minute, i.e., about 30 times more than at rest (Åstrand & Rodahl, 1986). Increasing the amount of air taken into to the respiratory system increases the quantity of the inhaled suspended particles matter, and thus its deposition in the respiratory track and in other organs of the body. This increases the risk and enhances the severity of several illnesses in humans (for review see, e.g., Bai, Li & Niu, 2020; Lauwers et al., 2020; Pope et al., 2019). Furthermore, knowledge concerning the quality of air can be very useful for patients suffering from cardio-pulmonary insufficiency, and who practice their physical rehabilitation program in the open air (for overview see, e.g., Dyer & Pugh, 2019).

Summing up, the practical outcome of our study can be expressed in the following three points: (i) the best hours to practice physical activities in Kraków in the open air, as judged by air quality, are between 10:00 and 14:00, whereas the worst time for practicing sports outdoors in Kraków is between 18:00 and 22:00; (ii) physical activities in the open air in Kraków during the winter (the season when the air is most polluted) should be limited and moved outside of the town; (iii) elderly people should consider the possibility of spending the winter outside of Kraków or at least to adjust the time spent in the open air in the town according to the air quality on a given day.

## Conclusions

We postulate that a low-cost air pollution monitoring system is capable of providing valuable information concerning the air quality in a given region, which seems to be of importance also to the people who practice varied sports activities in the open air.

##  Supplemental Information

10.7717/peerj.10041/supp-1 Supplemental Information 1 Raw data used for preparing Figure 4

## Additional Information and Declarations

### Data Availability

The following information was supplied regarding data availability: The raw measurements are available in the Supplementary File.
